# Production of *trans*-cinnamic acid by whole-cell bioconversion from l-phenylalanine in engineered *Corynebacterium glutamicum*

**DOI:** 10.1186/s12934-021-01631-1

**Published:** 2021-07-24

**Authors:** Jaewoo Son, Jun Hong Jang, In Hyeok Choi, Chang Gyu Lim, Eun Jung Jeon, Hyun Bae Bang, Ki Jun Jeong

**Affiliations:** 1 Department of Chemical and Biomolecular Engineering, BK21 Plus program, KAIST, 291 Daehak-ro, Yuseong-gu, Daejeon, 34141 Republic of Korea; 2 Institute for The BioCentury, KAIST, 291 Daehak-ro, Yuseong-gu, Daejeon, 34141 Republic of Korea

**Keywords:** Phenylalanine ammonia lyase, Whole-cell biocatalyst, Microfiltration membrane, Recycling reaction

## Abstract

**Background:**

*trans*-cinnamic acid (*t*-CA) is a phenylpropanoid with a broad spectrum of biological activities including antioxidant and antibacterial activities, and it also has high potential in food and cosmetic applications. Although significant progress has been made in the production of *t*-CA using microorganisms, its relatively low product titers still need to be improved. In this study, we engineered *Corynebacterium glutamicum* as a whole-cell catalyst for the bioconversion of l-phenylalanine (l-Phe) into *t*-CA and developed a repeated bioconversion process.

**Results:**

An expression module based on a phenylalanine ammonia lyase-encoding gene from *Streptomyces maritimus* (SmPAL), which mediates the conversion of l-Phe into *t*-CA, was constructed in *C. glutamicum*. Using the strong promoter P_H36_ and ribosome binding site (RBS) (in front of gene 10 of the T7 phage), and a high-copy number plasmid, SmPAL could be expressed to levels as high as 39.1% of the total proteins in *C. glutamicum*. Next, to improve *t*-CA production at an industrial scale, reaction conditions including temperature and pH were optimized; *t*-CA production reached up to 6.7 mM/h in a bioreactor under optimal conditions (50 °C and pH 8.5, using NaOH as base solution). Finally, a recycling system was developed by coupling membrane filtration with the bioreactor, and the engineered *C. glutamicum* successfully produced 13.7 mM of *t*-CA (24.3 g) from 18.2 mM of l-Phe (36 g) and thus with a yield of 75% (0.75 mol/mol) through repetitive supplementation.

**Conclusions:**

We developed a highly efficient bioconversion process using *C. glutamicum* as a biocatalyst and a micromembrane-based cell recycling system. To the best of our knowledge, this is the first report on *t*-CA production in *C. glutamicum*, and this robust platform will contribute to the development of an industrially relevant platform for the production of *t*-CA using microorganisms.

**Supplementary Information:**

The online version contains supplementary material available at 10.1186/s12934-021-01631-1.

## Background

*trans*-cinnamic acid (3-phenylpropenoic acid, *t*-CA) is a major phenolic compound in plants that acts as a precursor for various polyphenols, such as stilbenes and flavonoids [1]. Many studies have reported that *t*-CA has a broad spectrum of biological activities including antioxidant and antibacterial activities, and it also has high potential in food and cosmetic applications [2–5]. Moreover, since *t*-CA is regarded as a “generally recognized as safe (GRAS)” compound by the Food and Drug Administration (FDA), it could potentially have several uses; therefore, *t*-CA is attracting attention as an important substance to mankind [6]. Owing to the wide applicability of *t*-CA, research on the production of *t-*CA has been actively conducted in recent years. *t*-CA can be isolated from plants; however, this method has not yet overcome the low yield because it is difficult to optimize environmental and geographic conditions [7]. In addition, *t*-CA can be synthesized by the organic chemical synthesis method: a condensation reaction of acetic anhydride and benzaldehyde in the presence of sodium acetate [8–10]. Although the chemical synthesis accounts for the largest portion of *t*-CA production so far, it is an energy intensive and non-green process because it requires high temperature condition and fossil resources [11, 12]. Therefore, there is an urgent need for the development of more facile and safe methods such as biological methods; thus, microbial production of *t*-CA is drawing attention as an alternative strategy.

**Fig. 1:**
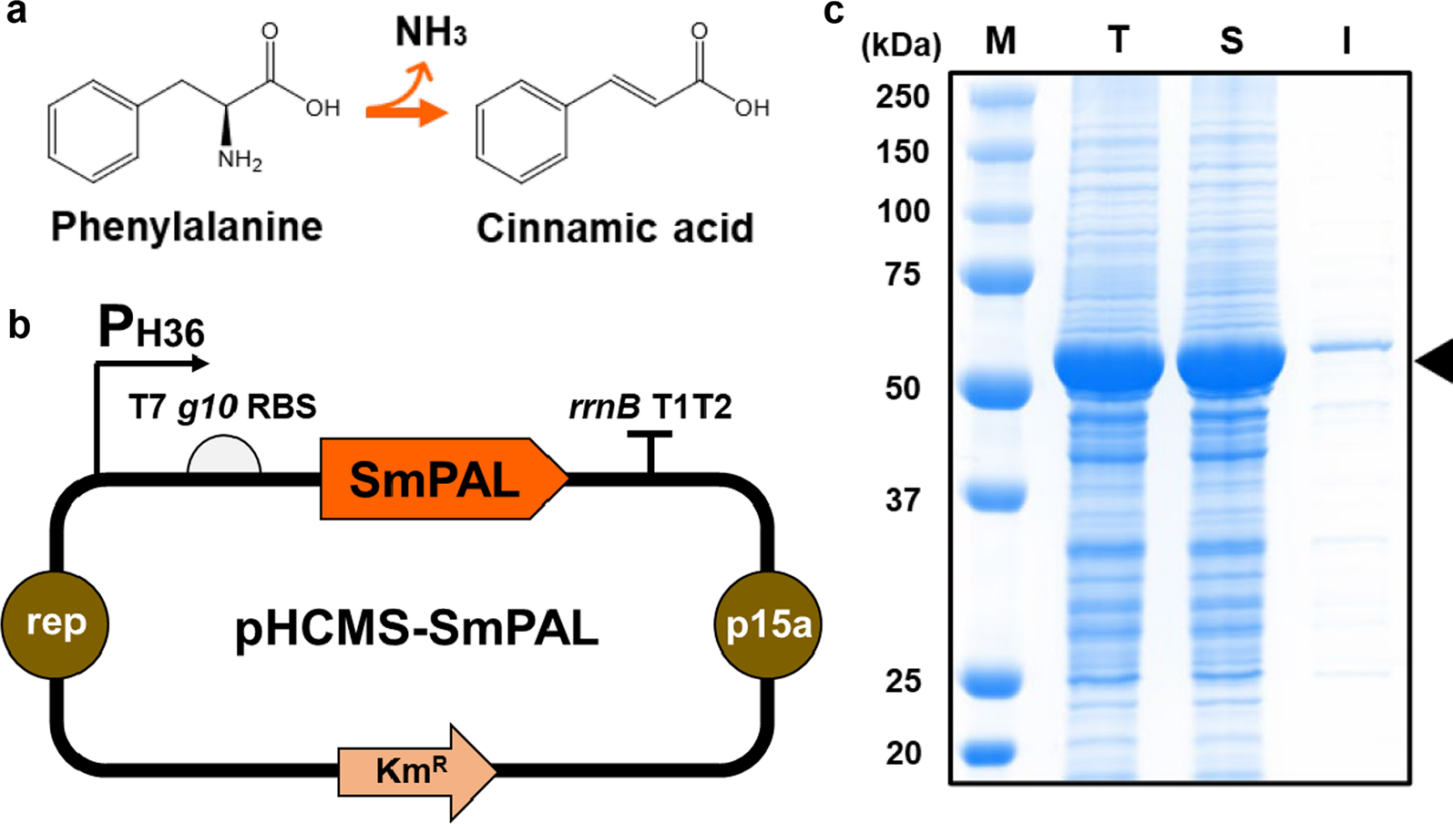
Expression of the gene encoding for SmPAL in *C. glutamicum* for *t*-CA production. **a** Schematic of conversion of l-Phe to *t*-CA by PAL. **b** Schematic diagram of the plasmid pHCMS-SmPAL constructed for the expression of the gene encoding for SmPAL in *C. glutamicum*. P_H36_, strong synthetic promoter; *rrnB T1T2*, transcriptional terminator; rep, replication origin of *C. glutamicum*; p15a, replication origin of *E. coli*. **c** SDS-PAGE analysis for the expression of the gene encoding for SmPAL in *C. glutamicum* harboring pHCMS-SmPAL. Lane M, molecular weight markers (kDa); Lane T, total protein fractions; Lane S, soluble protein fractions; Lane I, insoluble protein fractions. Arrowhead indicates the band of SmPAL

Naturally, *t*-CA can be synthesized from l-phenylalanine (l-Phe) by phenylalanine ammonia lyase (PAL, EC 4.3.1.5) [13]. PAL catalyzes the non-oxidative deamination of l-Phe and converts it to *t*-CA (Fig. 1a). In recent years, PAL has been of great interest in clinical, industrial, and biotechnological applications [14]. In particular, in the *t*-CA production method using microorganisms, PAL is recognized as an essential enzyme for synthesizing *t*-CA. Through recent evolution and diverse metabolic engineering strategies, a variety of microorganisms, such as *Escherichia coli*, *Streptomyces lividans*, *Saccharomyces cerevisiae*, and *Pseudomonas putida*, have been used to produce up to 0.8 g/L *t*-CA [11, 12, 15–18]. In addition, in a previous study, by expressing PAL in *E. coli* and optimizing cultivation conditions, we were able to produce 6.9 g/L of *t*-CA through fed-batch fermentation at a 2 L bioreactor scale [19]. To the best of our knowledge, this is the highest titer of *t*-CA production to date. However, although significant progress has been made in the production of *t*-CA using microorganisms, the relatively low productivity still needs to be improved.

Generally, compared to the chemical synthesis-based production process, the production of whole-cell biocatalyst-based chemicals using microorganisms offers several advantages: (i) environmentally friendly production, (ii) high regioselectivity, and (iii) cofactor regeneration for multi-step reactions [20]. However, to achieve a high production yield in whole-cell biocatalyst-based production, a microorganism that can satisfy the following conditions is required: (i) being recognized as a safe host for production, (ii) high-cell density cultures, (iii) the ability to easily induce high expression of the genes coding for heterologous proteins, and (iv) maintenance of cell viability during harsh conditions, e.g., changes in temperature or pH [21, 22]. In this respect, *Corynebacterium glutamicum* has been attracting attention as a bacterium suitable for use in a sustainable bio-based process using a whole-cell biocatalyst system. *C. glutamicum* is a non-sporulating and Gram-positive bacterium that has been used to produce l-amino acids, such as l-glutamate and l-lysine [23–25]. *C. glutamicum* has been widely used as an industrial strain because it can grow very densely in a limited space and thus obtain a high yield of cellular biomass concentrations compared to other bacteria. Furthermore, because *C. glutamicum* is generally recognized as safe (GRAS), the production of any compound of interest is also safe, reducing the limitations for downstream applications. In addition, recent advances in genetic engineering have enabled diverse applications of *C. glutamicum* as a potential industrial strain [26–29]. Considering these beneficial characteristics, *C. glutamicum* has been used as a whole-cell biocatalyst to produce various chemicals such as anthocyanin, keto-fatty acids, undec-9-enoic acid, and heptyl ester [30–35].

Here, we report the engineering of *C. glutamicum* as a whole-cell biocatalyst for the production of *t*-CA. First, we constructed an expression system for a PAL-encoding gene from *Streptomyces maritimus* (SmPAL) for the conversion of l-Phe to *t*-CA. Next, to improve the bioconversion into *t*-CA, the reaction conditions including temperature, pH, and base source were optimized. Finally, a crossflow microfiltration membrane module was coupled to the bioreactor, and recycled conversion of the whole-cell biocatalyst was performed with repeated addition of substrate (l-Phe) to achieve a high production yield for *t*-CA.

## Results

### Expression of the *SmPAL* gene in *C. glutamicum* for *t*-CA production

Previously, we examined the efficiencies of a few PAL enzymes for the biosynthesis of *t*-CA in *E. coli*, and SmPAL showed the highest production of *t*-CA [36]. Moreover, unlike other PALs, SmPAL is also known to have relatively low TAL (tyrosine ammonia lyase) activity leading to *p*-coumaric acid instead of *t*-CA [37, 38]. Hence, the used enzyme is suitable for reducing side product formation during the bioconversion process. Based on those characteristics, we decided to employ SmPAL to synthesize *t*-CA in *C. glutamicum* and constructed an expression system for this gene. In the whole-cell biocatalyst system, the *t*-CA bioconversion rate can be correlated with the content of SmPAL in a cell; therefore, we used the high-copy-number plasmid pHCMS [39] to clone the *SmPAL* gene under the control of the strong synthetic constitutive promoter P_H36_ [40] (Fig. 1b). In addition, the ribosome binding site (RBS) (in front of gene 10 of the T7 phage), which is known to promote efficient gene expression in *C. glutamicum*, was also introduced because, irrespective of the transcription start site strength, the untranslated region (UTR) sequence between the promoter and start codon of the *SmPAL* gene can affect gene expression in *C. glutamicum* (Fig. 1b). Using this construct (pHCMS-SmPAL), we confirmed by SDS-PAGE that SmPAL (56 kDa) was successfully synthesized in *C. glutamicum* (Fig. 1c). Most SmPAL was produced in soluble form, and its maximum content was 39.1% of the total protein amount (Fig. 1c).

### Evaluation of the bioconversion into *t*-CA with engineered *C. glutamicum*

**Fig. 2:**
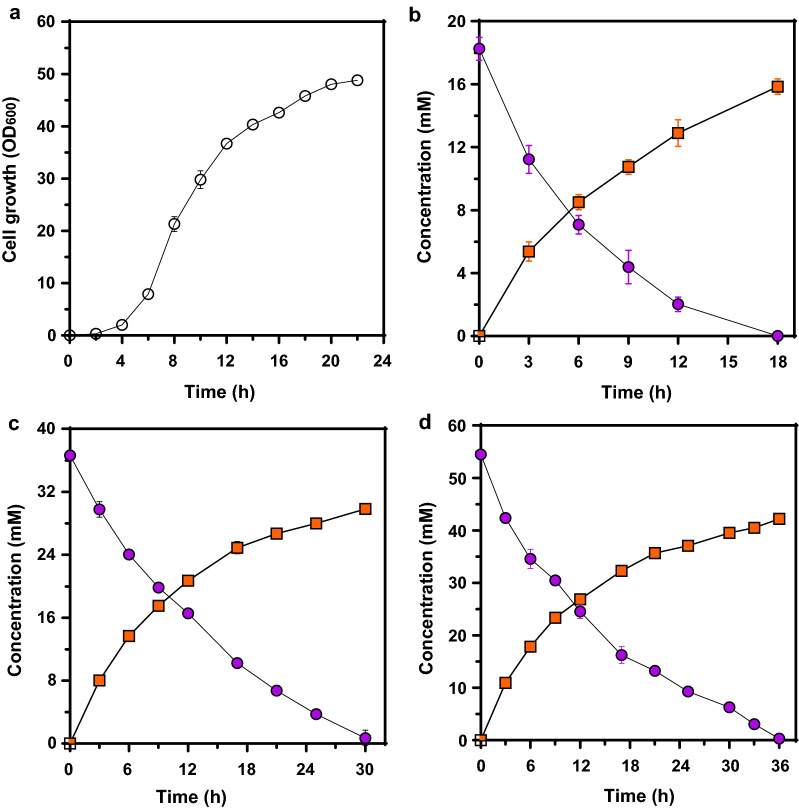
Production of *t*-CA from l-Phe in a flask cultivation. **a** Time profile of cell growth in optical density (OD) at 600 nm. Symbol: open circle, *C. glutamicum* harboring pHCMS-SmPAL. **b** 18.2 mM of l-Phe, **c** 36.3 mM of l-Phe, **d** 54.5 mM of l-Phe. **b–d** Symbols: closed circle, l-Phe; closed square, *t*-CA. Results are the mean of duplicate experiments and error bars indicate standard deviations

With *C. glutamicum* harboring pHCMS-SmPAL, the bioconversion of l-Phe to *t*-CA was evaluated in flask scale cultures. In shaking flask cultures, the cells were cultivated at 30 ℃ and reached the stationary phase after 22 h (Fig. 2a). 18.2 mM of l-Phe (3 g/L) was then added, and the bioconversion of l-Phe to *t*-CA was immediately observed (Fig. 2b). For 18.2 mM of l-Phe, complete consumption of the substrate was observed after approximately 18 h, and we found that 15.9 mM of *t*-CA had been synthesized with a conversion rate of 0.8 mM/h and a yield of 87% (0.87 mol/mol) (Fig. 2b). Next, the bioconversion efficiencies were evaluated using higher concentrations of l-Phe (36.3 and 54.5 mM). When a concentration of 36.3 mM of l-Phe (6 g/L) was used, the substrate was completely consumed in 30 h, and 29.7 mM of *t*-CA was produced with a conversion yield of 81% (0.81 mol/mol) (Fig. 2c). In addition, when using 54.5 mM of l-Phe (9 g/L), total consumption was reached after 36 h, and 42.5 mM of *t*-CA was produced with a conversion yield of 78% (0.78 mol/mol) (Fig. 2d). Although it was clearly confirmed that the engineered cells could convert l-Phe into *t*-CA, resulting in high yields, the conversion rates were not high enough, so we sought to further optimize the reaction conditions.

### Enhancing conversion rate for *t*-CA by increasing reaction temperature

**Fig. 3:**
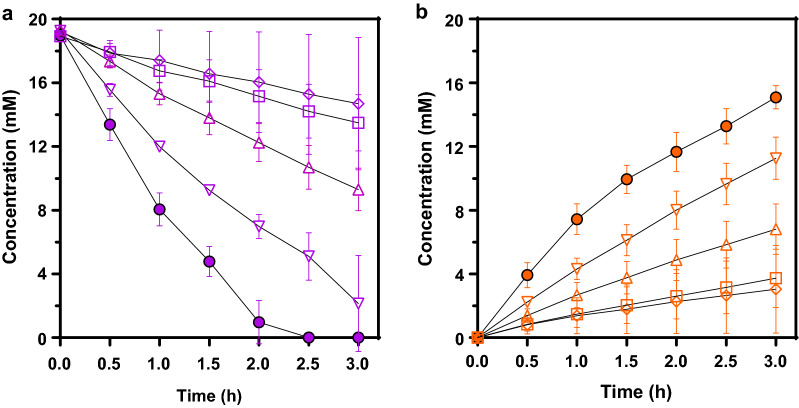
Evaluation of conversion rate for *t*-CA at various reaction temperature. **a** Concentration of l-Phe. Symbols: open square, 30 ℃; open triangle up, 37 ℃; open triangle down, 45 ℃; closed circle, 50 ℃; open diamond, 55 ℃. **b** Concentration of *t*-CA. Symbols: same as **a**. Purple indicates concentration of l-Phe and orange indicates concentration of *t*-CA. Results are the mean of duplicate experiments and error bars indicate standard deviations

Previously, it was reported that the activity of PAL enzymes derived from *Musa* spp., *Triticum aestivum*, *Ephedra sinica*, and *Vitis vinifera* increased rapidly as the reaction temperature increased [41]. Therefore, we compared the conversion rate of l-Phe to *t*-CA catalyzed by SmPAL at different reaction temperatures (30, 37, 45, 50, and 55 ℃). The cells were cultivated in flasks at 30 ℃ during the exponential growth phase and, immediately after reaching the stationary phase, the cells were further incubated for 30 min to reach each target temperature. Subsequently, 18.2 mM l-Phe was added, and the amount of *t*-CA produced per time unit was analyzed. Overall, the conversion rate rapidly increased as the reaction temperature increased (Fig. 3a, b). In particular at 50 ℃, complete consumption of the entire 18.2 mM l-Phe was observed at 2.5 h, and the reaction rate increased by about 4 times compared to that at 30 ℃ (Table 1). For 18.2 mM l-Phe, *t*-CA produced at 3 h was observed to be 15.3 mM (Fig. 3b), and there was no significant difference compared with the *t*-CA final yield when the substrate was completely consumed at 30 ℃ reaction (18 h) (Fig. 2b). Therefore, it was confirmed that the increasing reaction temperature contributes to enhance the conversion rate but does not affect *t*-CA production yield. Meanwhile, a sharp decrease in the reaction rate was observed at 55 °C (Fig. 3a, b). Based on these data, we decided to set the temperature at 50 °C for performing reaction in a bioreactor.

**Table 1:** Summary of *t*-CA production at different temperatures in *C. glutamicum* harboring pHCMS-SmPAL

Reaction temperature (°C)	Concentration^a^ (mM)	Conversion rate^b^ (mM/h)
30	3.2 ± 1.7	1.3 ± 0.7
37	5.9 ± 1.5	2.3 ± 0.6
45	9.6 ± 1.3	3.9 ± 0.5
50	13.2 ± 1.1	5.3 ± 0.4
55	2.6 ± 2.3	1.0 ± 0.9

^a,b^Concentration and conversion rate of *t*-CA were analyzed at 2.5 h. Data are summarized as mean ± standard deviation (n = 2)

^b^Conversion rate is the total rate calculated at 2.5 h of the whole reactions

### Bioconversion into *t*-CA with engineered *C. glutamicum* in a bioreactor

**Fig. 4:**
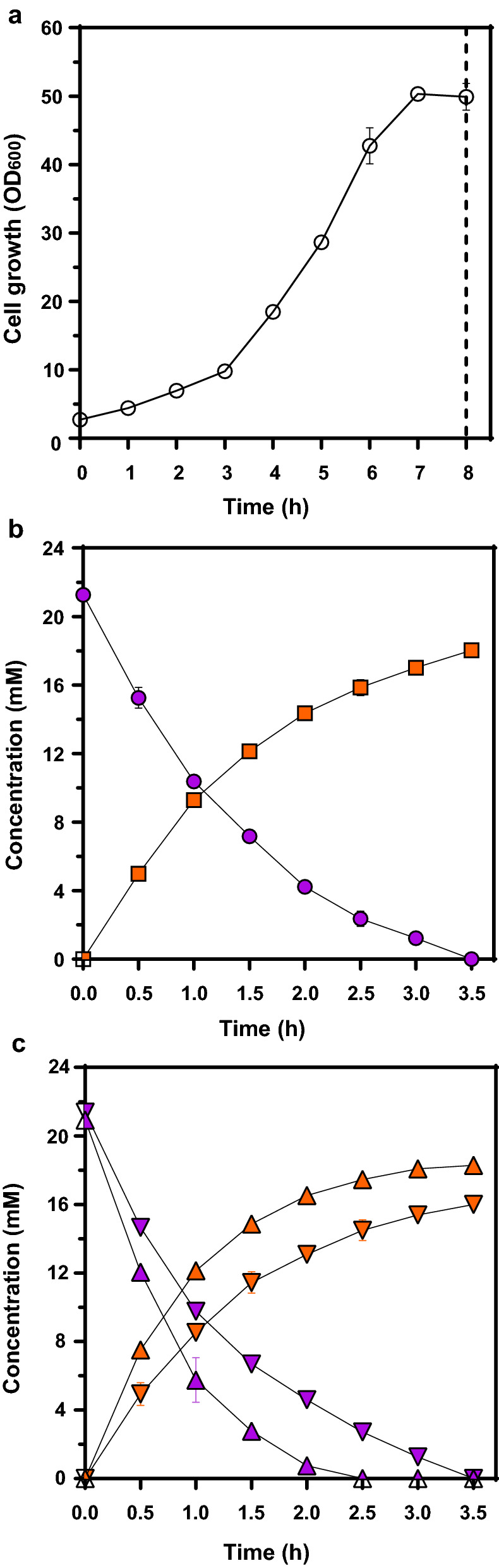
Evaluation of bioconversion into *t*-CA at a bioreactor scale (2 L). **a** Time profile of batch cultivation. Symbols: open circle, optical density (OD) at 600 nm of *C. glutamicum* harboring pHCMS-SmPAL. The dashed line indicates the induction point. **b** Bioconversion profile at pH 7.5. Symbols: closed circle, l-Phe; closed square, *t*-CA. **c** Optimization of pH for enhancing conversion efficiency for *t*-CA with different base solutions. Symbols: closed triangle down, reaction at pH 8.5 with 5 M ammonia salutation; closed triangle up, reaction at pH 8.5 with 10 M NaOH. **b **and **c** Purple indicates concentration of l-Phe and orange indicates concentration of *t*-CA. Results are the mean of duplicate experiments and error bars indicates standard deviations

Next, to confirm the feasibility of the developed whole-cell biocatalyst on a large scale, the performance of *C. glutamicum* for the conversion of l-Phe to *t*-CA was evaluated in a 2 L bioreactor. Cells were incubated at 30 °C and grown to an OD_600_ of 50 after 7 h (Fig. 4a). When the cells reached the stationary phase, they were further incubated for 1 h to reach 50 °C, and the pH was adjusted to 7.5 by adding 5 M ammonia solution. Then, 21.2 mM of l-Phe (3.5 g/L) was added for the bioconversion reaction to *t*-CA. As shown in Fig. 4b, the added l-Phe was rapidly converted to *t*-CA in the bioreactor. Complete consumption of l-Phe was observed at approximately 3.5 h; at that time, the converted *t*-CA concentration was 17.5 mM, indicating a production rate of 5.0 mM/h (Fig. 4b). Because complex ingredients including casamino acids and BHI were supplied in the cultivation, *t*-CA can be synthesized using the residual l-Phe during conversion reaction irrespective of l-Phe supplementation, which might be included in the yield analysis. For accurate yield analysis, we performed same bioconversion reaction without supplementation of l-Phe, and we found that the *t*-CA titer was approximately 0.4 mM (Additional file 1: Figure S1), which was negligible compared with that under supplementation of l-Phe. Thus, it was confirmed that the *t*-CA titer observed in Fig. 4b was mostly derived from the reaction with added substrate.

In addition, we compared the reaction rates in relation to changes in pH in the large-scale bioreactor. Previous studies have confirmed that PAL is active at a pH between 6.5 and 9.0, and that SmPAL shows maximum activity at pH 8.5 [37]. In the flask cultures of *C. glutamicum* harboring pHCMS-SmPAL, we also confirmed that the reaction rate gradually increased as the pH increased from 6.5 to 8.5 (Additional file 2: Figure S2). Based on these flask results, we performed the conversion reaction in a bioreactor at pH 8.5 to ensure the highest conversion efficiency. After cultivation of cells at pH 7.0 and 30 °C, the temperature was increased to 50 °C and the pH of the culture medium was increased to pH 8.5 by adding 5 M ammonia solution. However, contrary to the results from flasks, the reaction rate at pH 8.5 (4.3 mM/h) was lower than that at pH 7.5 (5.0 mM/h) in the bioreactor (Fig. 4c). This was a different result from those in previous studies [37]. Maldonado et al. reported that the presence of ammonia in the reaction reduced the relative activity of PAL extracted from cherimoya to a level of 33% compared to the normal condition [42]. So, we hypothesized that ammonia which was used for pH control shifted the equilibrium to the substrate side (l-Phe) in the conversion of l-Phe to *t*-CA through the PAL enzyme. Therefore, instead of the 5 M ammonia solution, we used a 10 M NaOH solution to control the pH and observed that l-Phe was completely consumed in 2.5 h, which was approximately 1 h faster than when using ammonia solution (either at pH 8.5 or 7.5), and the conversion rate increased to 6.7 mM/h (Fig. 4c).

Next, we analyzed the conversion efficiencies when higher concentrations of l-Phe were added to the media, including 33.3 mM, 42.4 mM, and 54.5 mM. When the concentration of l-Phe was 33.3 mM, complete consumption of the amino acid was observed in 9 h, and 27.7 mM of *t*-CA was produced with a yield of 83% (0.83 mol/mol) (Additional file 3: Figure S3). However, at higher concentrations of l-Phe, bioconversion was stopped without complete consumption of the substrate. For a concentration of 42.4 mM of l-Phe, 28.3 mM of *t*-CA was produced after 10 h with a yield of 66% (0.66 mol/mol) and the reaction terminated with a remnant of 7.3 mM of l-Phe (Additional file 3: Figure S3). Moreover, for a concentration of 54.5 mM of l-Phe, 43.2 mM of *t*-CA was produced after 10 h with a yield of 79% (0.79 mol/mol) and the reaction was terminated with 10.9 mM of l-Phe remaining (Additional file 3: Figure S3). These results suggest that the conversion of high concentrations of l-Phe to *t*-CA through a single batch reactor is not the appropriate method to achieve high production yield. Thus, it is necessary to develop a new method.

### Production of *t*-CA using a crossflow membrane-based cell recycling system

**Fig. 5:**
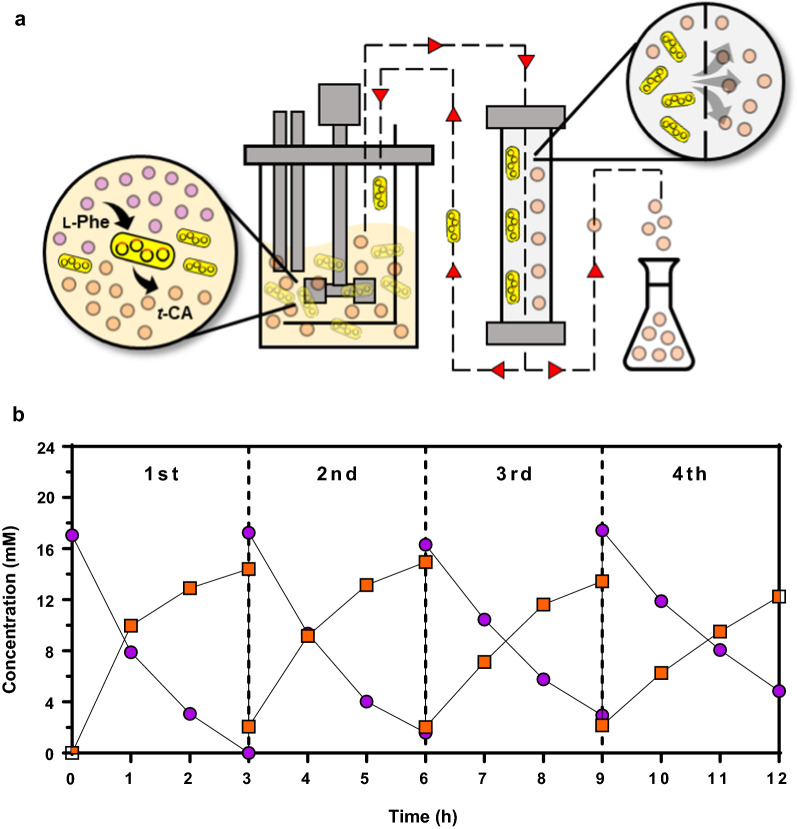
Repeated conversion with *C. glutamicum* harboring pHCMS-SmPAL using microfiltration membrane module at a bioreactor scale. **a** Schematic diagram of module for repeated production of *t*-CA with microfiltration membrane module. **b** Time profiles of production titer of *t*-CA. Symbols: closed circle, l-Phe; closed square, *t*-CA

To perform the bioconversion reaction with a high amount of substrate, we developed a crossflow membrane-based cell recycling system in which the bioreactor and a crossflow microfiltration membrane module were coupled. In this system, l-Phe (18.2 mM) was repeatedly supplied to the bioreactor, while produced *t*-CA was removed by crossflow membrane filtration and cells were circulated to the bioreactor for the next round of reaction (Fig. 5a). Compared with the one-batch reaction, more l-Phe could be supplied for bioconversion, and using the membrane filtration, the synthesized *t*-CA was periodically removed from the reactor, by which accumulation of toxic product (*t*-CA) in the bioreactor could be prevented. In the first round, supplied l-Phe (18.2 mM) was completely consumed, and 14.2 mM of *t*-CA was successfully synthesized (Fig. 5b). The bioconversion reaction was further repeated three more times, and as shown in Fig. 5b, the production of *t*-CA was maintained at the similar level throughout the four cycles, suggesting that the repeated cycle also did not deteriorate the integrity of the cells. In four cycles, total 18.2 mM of l-Phe (36 g) was consumed and 13.7 mM of *t*-CA (24.3 g) was produced with a yield of 75% (0.75 mol/mol). The entire process from cell cultivation to four-cycle conversion reactions was completed in 20 h, and a productivity as high as 0.7 mM/h could be achieved.

## Discussion

*t*-CA may be widely applied in health, food, and pharmaceutical sectors. Its production using microorganisms can be an attractive alternative to organic chemical synthesis, which causes environmental pollution problems. For the enhanced production of *t*-CA, we engineered *C. glutamicum* as whole-cell biocatalyst, and a high production yield could be achieved through optimization of the bioconversion reaction in a bioreactor coupled with cell recycling and membrane filtration systems. In an earlier study, we engineered *E. coli* for the production of *t*-CA and obtained up to 6.9 g/L of *t*-CA by fed-batch cultivation with an engineered strain [19]. Although this titer was the highest ever recorded, the productivity was not high (0.138 g/L/h) because of the long cultivation (86 h) [19]. For improving this time, a modification in the reaction temperature was considered. As shown here, SmPAL showed its highest activity at 50 °C; an activity four times higher than that at 30 °C (Fig. 3). Unfortunately, *E. coli* and most bacterial hosts cannot be cultivated at 50 °C but are generally grown between 30 and 37 °C at which the activity of the PAL enzyme is not high enough. This temperature difference is one of the hurdles to be solved in the production of *t*-CA using microorganisms. In contrast to *E. coli*, *C. glutamicum* has a very rigid cell wall structure, which consists of mycolic acid, arabinogalactan, and a peptidoglycan layer [43]. Due to this distinct structure, *C. glutamicum* is known for its high tolerance to various chemicals and extreme conditions such as high temperature, pH, and detergents, so it has served as a robust and potential platform for the production of toxic chemicals and production under extreme conditions [44–46]. Although *C. glutamicum* cells were not alive at 50 °C, *C. glutamicum* cells were not easily disrupted due to the robust cell wall structure, and SmPAL which is still active at 50 °C, is not released at this temperature condition. Using this rigid host, bioconversion reactions to *t*-CA could be conducted at 50 °C, and a much higher conversion rate (5.3 ± 0.4 mM/h) could be achieved compared with that obtained using *E. coli* (Table 1). In addition, because of its high tolerance to high temperatures, the *C. glutamicum* platform could be coupled to a recycling system, and cells consistently showed *t-*CA production capability even at all four consecutive rounds of reactions at 50 °C. In repeated conversion reactions, however, we also found that the *t*-CA production yield decreased gradually (Fig. 5b), and this may be ascribed to the loss of activity of SmPAL. High temperature and pH conditions to achieve maximum production yield can conversely reduce the stability of the PAL enzyme. In a previous study, it was reported that the stability of PAL was reduced by 4 times or more under 50 °C compared to 30 °C [47], thus, to increase the reusability of the current system, engineering of PAL with high enzymatic activity even at low temperature or high stability at high temperature can be considered, which will be our next work.

In all bioconversion reactions, *C. glutamicum* showed a high potential as a whole-cell biocatalyst for the production of *t*-CA, but we could not achieve 100 % conversion yield. One reason might be the possible activation of the catabolic pathway related to the degradation for phenylpropanoids inherent in *C. glutamicum*. In *C. glutamicum*, the *phd* gene cluster was identified to be involved in the utilization of phenylpropanoids via the CoA-dependent, β-oxidative deacetylation route [48]. Although the transcriptional regulator PhdR for the activation of the *phd* gene cluster is not reactive with *t*-CA [48], it may be activated by other phenylpropanoids such as *p*-coumaric acid, caffeic acid, and ferulic acid which are derived from amino acids l-tyrosine and l-Phe, and consequently the synthesized *t*-CA can be degraded [48]. In this work, even though we performed a reaction at 50 °C, at which cells were not alive, selective removal of the phenylpropanoid degradation pathway can be considered as a strategy to further increase the production efficiency for *t*-CA.

In the development of whole-cell biocatalysts, the expression of genes encoding key enzymes is also critical. In most fermentation processes, the expression levels of genes encoding key enzymes need to be optimized to reduce the metabolic burden on the production host: too high expression of genes encoding key enzymes may cause poor cell growth, consequently, the production titer of target materials decreases [49]. However, in whole-cell biocatalyst platforms, we do not need to consider the metabolic burden in the hosts, so the expression level can be increased to the highest level, and an efficient bioconversion can be achieved. In this regard, the use of *C. glutamicum* as a whole-cell catalyst showed beneficial results compared to those from the *E. coli* fermentation processes. Previously, our team developed many useful synthetic tools for gene expression in *C. glutamicum*, including synthetic promoters, RBS, and high-copy number plasmids [39, 40]. In the present study, all these tools were considered for optimizing the expression of the gene coding for SmPAL, a key enzyme in bioconversion into *t*-CA. Placing the *SmPAL* gene under the control of the strong promoter P_H36_ and the RBS (in front of gene 10 of the T7 phage) in a high-copy-number plasmid, the SmPAL content reached 39.1% of total proteins of the *C. glutamicum* strain used as host; this content was much higher than that obtained in *E. coli* under the control of the strong IPTG inducible promoter P_trc_ [19].

In addition to the expression level, the solubility of an enzyme is critical for enzyme reactions in the host. Previously, the aggregation of SmPAL into insoluble inclusion bodies was detected when the gene was expressed in *E. coli* even under the control of different promoters (P_tac_, P_trc_, and P_BAD_). This aggregation caused a reduction in both enzymatic activity and production titers. In contrast, although the expression level of the *SmPAL* gene was high in *C. glutamicum*, most SmPAL enzymes were not aggregated into inclusion bodies but were produced in a highly soluble form (Fig. 1c), which consequently allowed a highly efficient enzymatic reaction. According to the much higher expression level of the *SmPAL* gene and solubility of SmPAL, *C. glutamicum* can be used as a potential host for the production of *t-*CA.

## Conclusions

In this study, we developed a highly efficient bioconversion process using *C. glutamicum* as a biocatalyst. With a high-level gene expression system, the reaction condition was optimized (i.e. 50 °C and pH 8.5, using NaOH as a base solution), and by using a recycling system coupled with membrane filtration, we could achieve much improved productivity (0.7 mM/h). To the best of our knowledge, this is the first report on *t*-CA production in *C. glutamicum*, whereas the productivity is also the highest recorded in microbial production systems to date [19]. Compared with microbial fermentation processes which require much longer cultivation times (~ several days) and have the limit in the production titer due to the toxicity of *t*-CA [12, 19], our bioconversion system with the robust *C. glutamicum* is highly competitive and represents a highly stable and cost-effective platform. By engineering the key enzyme (i.e. SmPAL) toward higher activity and establishing the optimal downstream process for purification of the product, the overall process can be further improved, and we believe our bioconversion system can be a major contributor to the commercial production of *t*-CA in the bioindustry.

## Materials and methods

### Bacterial hosts and culture conditions

The bacterial strains and plasmids used in this study are listed in Table 2. *E. coli* XL1-Blue was used for gene cloning and plasmid maintenance, and *C. glutamicum* ATCC 13,032 was used as the main host for bioconversion. *E. coli* XL1-Blue was cultivated in Luria-Bertani medium (BD, Franklin Lakes, NJ, USA) at 37 ℃ and 200 rpm. *C. glutamicum* was cultivated in CGXII medium (3 g/L K_2_HPO_4_, 1 g/L KH_2_PO_4_, 2 g/L urea, 10 g/L (NH_4_)_2_SO_4_, 2 g/L MgSO_4_, 200 µg/L biotin, 5 mg/L thiamine, 10 mg/L calcium pantothenate, 10 mg/L FeSO_4_, 1 mg/L MnSO_4,_ 1 mg/L ZnSO_4_, 200 µg/L CuSO_4_, 10 mg/L CaCl_2_ and 20 g/L glucose) [50] supplied with 15 g/L brain heart infusion (BHI [BD]) and 7 g/L casamino acid. Kanamycin (25 µg/mL) was added to all culture media as the sole antibiotic.

**Table 2:** Bacterial strains and plasmids used in this study

Strains/plasmids	Description	References
*E. coli* XL1-Blue	*recA1 endA1 gyrA96 thi*-*1 hsdR17 supE44 relA1 lac [F´ proAB lacIqZDM15 Tn10 (Tetr)]*	Stratagene^a^
﻿*C . glutamicum* ATCC 13032	The wild-type strain	ATCC
Plasmids		
pHCMS	pCES-PLPV derivative; *parB* nonsense mutation, Km^R^	[39]
pHB-104	pTak15k derivative, P_tac_, *SmPAL*	[36]
pHCMS-SmPAL	pHCMS derivative; P_H36_, RBS (in front of gene 10 of the T7 phage), *SmPAL*, Km^R^	This study

^a^Stratagene Cloning Systems, La Jolla, CA, USA

### Plasmid construction

For gene expression in *C. glutamicum*, pHCMS, the high-copy number plasmid derivative of pCES208, was used as the backbone plasmid [39]. Polymerase chain reaction (PCR) was performed using a C1000TM Thermal Cycler (Bio-Rad, Richmond, CA, USA) and PrimeSTAR HS DNA Polymerase (Takara Bio Inc., Shiga, Japan). To express SmPAL, the native *SmPAL* gene was amplified from pHB-104 [36] by PCR using primers F-T7R-SmPAL and R-SmPAL (Additional file 4: Table S1). The PCR product was digested with restriction enzymes *Xba*I and *Not*I and cloned into pHCMS, yielding pHCMS-SmPAL, in which the *SmPAL* gene was constitutively expressed under the strong synthetic promoter P_H36_ [40].

### Protein preparation and analysis

After culturing at 30 ℃ for 12 h in a shake-flask, cells were harvested by centrifugation (6000 rpm, 10 min, 4 ℃) and washed with phosphate-buffered saline (PBS; 135 mM NaCl, 2.7 mM KCl, 4.3 mM ${\text{Na}}_{{2}}{\text{HPO}}_{{4}}$, pH 7.2). Then, cells were disrupted by sonication (7 min with 5-s pulse and 3-s cooling time, 20% amplitude). After collecting the total fractions, insoluble pellets were eliminated by centrifugation (13,000 rpm, 5 min, 4 ℃), and the soluble fraction was collected from the supernatant. For SDS-PAGE analysis, the protein samples were loaded onto a 12% polyacrylamide gel. After electrophoresis, the gel was stained with Coomassie brilliant blue (50% [*v/v*] methanol, 10% [*v/v*] acetic acid, and 1 g/L Coomassie brilliant blue R-250) for 1 h and then destained with a destaining solution (10% [*v/v*] methanol, 10% [*v/v*] acetic acid). Densitometry analysis was performed using the Image Lab software (Bio-Rad).

### Whole-cell bioconversion in a shake flask

To analyze *t*-CA production at 30 °C, *C. glutamicum* harboring pHCMS-SmPAL was inoculated into BHI (BD) medium. After 12 h, cells were transferred into 50 mL of semi-defined medium in 250 mL baffled flasks at 1:100 dilution and grown at 30 ℃ with shaking (200 rpm). Cell growth was determined by measuring the optical density at 600 nm (OD_600_) using a spectrophotometer (Optizen POP; Mecasys, Daejeon, Republic of Korea). After the cells reached the stationary phase, they were harvested by centrifugation at 6000 rpm for 10 min at 4 ℃. After harvesting, the cells were resuspended in 50 mL of 100 mM Tris-HCl buffer at pH 7.5 and incubated for 30 min in a shaking incubator to reach 30 ℃. Then, 25 mL of 100 mM Tris-HCl buffer pH 7.5 containing 225 mg of l-Phe pre-heated to 30 ℃ was added to the cells. All conversion reactions in the shake flask were performed at 200 rpm in a shaking incubator.

To compare the conversion rate at various reaction temperatures, after harvesting cells were resuspended in 50 mL of 100 mM Tris-HCl buffer (pH 7.5) and incubated for 30 min in a shaking incubator to reach target temperatures. Then, 25 mL of 100 mM Tris-HCl buffer pH 7.5 containing 225 mg of l-Phe pre-heated to the corresponding target temperature was added to the cells.

To evaluate the conversion rate at various pH conditions, after cells were harvested under the same conditions as above, they were resuspended in 50 mL of 100 mM Tris-HCl buffer at different target pH values. Then, they were incubated for 30 min in a shaking incubator to reach 30 ℃. Then, 25 mL of 100 mM Tris-HCl buffer at the corresponding pH containing 225 mg of l-Phe, pre-heated to 30 ℃ was added to the cells.

### Whole-cell bioconversion in a bioreactor

*Corynebacterium glutamicum* harboring pHCMS-SmPAL was inoculated into BHI (BD) medium and cultured for 12 h; then, cells were transferred into 200 mL of semi-defined medium in four 250 mL baffled flasks (50 mL in each flask). All seed cultures were poured into 1.8 L of fresh semi-defined medium in a 5 L jar bioreactor (BioCNS, Daejeon, Republic of Korea). Culture temperature was set to 30 ℃ until the cells reached the stationary phase. While cells were growing, pH was adjusted to pH 7, either with sulfuric acid (when the pH was above 7.06) or 5 M ammonia solution or 10 M NaOH (when the pH was below 6.97), with online monitoring. After the cells reached the stationary phase, the temperature was increased to 50 ℃ and the pH was adjusted to either pH 7.5 or pH 8.5. Then, 1 L of 100 mM Tris-HCl buffer at pH 7.5 or pH 8.5 containing 10.5 g of l-Phe pre-heated to 50 ℃ was added to the bioreactor.

### High performance liquid chromatography (HPLC) analysis

To measure the amount of l-Phe and *t*-CA, cells were pelleted by centrifugation at 13,000 rpm for 5 min at 4 °C, and the supernatants were filtered through 0.22 μm syringe filters (Futecs, Daejeon, Korea). Filtered supernatants were diluted 1:10 in 10% (*v/v*) acetonitrile and analyzed by HPLC. The HPLC system (Shimadzu, Kyoto, Japan) consisted of pump (LC-20AD), autosampler (SIL-30AC), column oven (CTO-20 A), and refractive index detector (RID-10 A), and was equipped with a Zorbax Eclipse AAA column (150 × 4.6 mm, 3.5 microns; Agilent Technologies, PA, CA, USA). Samples were separated using a binary nonlinear gradient with mobile phase A (0.1% [*v/v*] trifluoroacetic acid [TFA]) and mobile phase B (acetonitrile). The column temperature was maintained at 40 °C and a 1 mL/min flow rate. For l-Phe analysis, elution conditions were as follows: (i) equilibrate the column with 10% mobile phase B for 6 min, (ii) run gradient from 10 to 70% mobile phase B for 4 min, (iii) maintaining flow at 70% mobile phase B for 7 min, (iv) run gradient from 70 to 10% mobile phase B for 3 min, and (v) wash with 10% mobile phase B for 5 min. The samples were detected using a UV detector at 280 nm.

For *t*-CA analysis, elution conditions were as follows: (i) equilibrate the column with 10 % mobile phase B for 1 min, (ii) run gradient from 10 to 70% mobile phase B for 19 min, (iii) run gradient from 70 to 10% mobile phase B for 5 min, and (iv) clean with 10% mobile phase B for 3 min. Chromatographic software Lab Solution (Shimadzu, Kyoto, Japan) was used for sample acquisition and data analysis. The samples were detected using a UV detector at 210 nm.

### Conversion using a membrane-based cell recycle module

Seed culture preparation followed the same procedure as that for bioreactor cultures. Then, seed cultures were poured into 1.8 L of fresh semi-defined medium in a 5 L jar bioreactor (BioCNS). The cultivation temperature was set at 30 ℃ until the cells reached the stationary phase. While the cells were growing up, pH was adjusted to pH 7 with 10 M NaOH (when the pH was below 6.97) with online monitoring. After the cells reached the stationary phase, the temperature was increased to 50 ℃ and the pH was adjusted to pH 8.5 with 10 M NaOH. Then, 1 L of 100 mM Tris-HCl pH 8.5 buffer containing 9 g of l-Phe pre-heated to 50 ℃ was added to the bioreactor.

A 5 L bioreactor (BioCNS) was used for repeated conversion, with a working volume of 3 L. The bioreactor was connected to a crossflow microfiltration membrane module (Labio 203, 0.1 μm; Philos, Gwangmyeong, Korea). An inlet line from the bioreactor, an outlet line to the bioreactor, and a permeate line from the membrane module were installed (Fig. 5a). A pressure gauge was attached to the permeate line to monitor the transmembrane pressure and keep it under 40 mmHg. The air line from the compressor was attached to the module inlet. A 5 L product vessel was assembled to the system to collect the permeate. The fermentation broth was circulated through the microfiltration membrane module at 380 mL/min and removed using two peristatic pump models (Cole-Parmer Instrument, Vernon Hills, IL, USA). After each run, the microfiltration membranes were cleaned and sterilized with 300 ppm NaOCl. Sterilized deionized water was used to flush the module to remove the residual NaOCl solution. Before each experiment, the bioreactor, feed vessel, and product vessel were autoclaved at 121 ℃ for 15 min.

## Supplementary Information

**Additional file 1: Figure S1.** Evaluation of *t*-CA titer by the amount of additional l-Phe present in complex ingredients of cell culture medium.

**Additional file 2: Figure S2.** Evaluation of conversion rate for *t*-CA at various pH conditions in a flask cultivation.

**Additional file 3: Figure S3.** Evaluation of bioconversion into *t*-CA at a bioreactor scale (2 L) with various concentrations of l-Phe as a substrate.

**Additional file 4: Table S1.** Oligonucleotides used in this study.

## Data Availability

Please contact corresponding author for data requests.

## Abbreviations

l-Phe: l-phenylalanine
*t*-CA: *trans*-cinnamic acid
PAL: Phenylalanine ammonia lyase
SmPAL: Phenylalanine ammonia lyase from *Streptomyces maritimus*
RBS: Ribosome binding sites
IPTG: Isopropyl-β-d-thiogalactopyranoside
