# Supplementary material for: Production of trans-cinnamic acid by whole-cell bioconversion from l-phenylalanine in engineered Corynebacterium glutamicum
Source: Microb Cell Fact. 2021 Jul 24;20:145. doi: 10.1186/s12934-021-01631-1 (PMC8310591; doi:10.1186/s12934-021-01631-1)
Supplement: Supplementary file 1 — Additional file 1: Figure S1. Evaluation of t-CA titer by the amount of additional l-Phe present in complex ingredients of cell culture medium. [file 12934_2021_1631_MOESM1_ESM.docx]

**Figure S1** Evaluation of *t*-CA titer by the amount of additional L-Phe present in complex ingredients of cell culture medium. Reaction was performed at pH 7.5 (5 M ammonia solution) and 50 °C in a bioreactor (2 L). Symbols: closed square, concentration of *t*-CA. Results are the mean of duplicate experiments.
