# Supplementary material for: Production of trans-cinnamic acid by whole-cell bioconversion from l-phenylalanine in engineered Corynebacterium glutamicum
Source: Microb Cell Fact. 2021 Jul 24;20:145. doi: 10.1186/s12934-021-01631-1 (PMC8310591; doi:10.1186/s12934-021-01631-1)
Supplement: Supplementary file 2 — Additional file 2: Figure S2. Evaluation of conversion rate for t-CA at various pH conditions in a flask cultivation. [file 12934_2021_1631_MOESM2_ESM.docx]

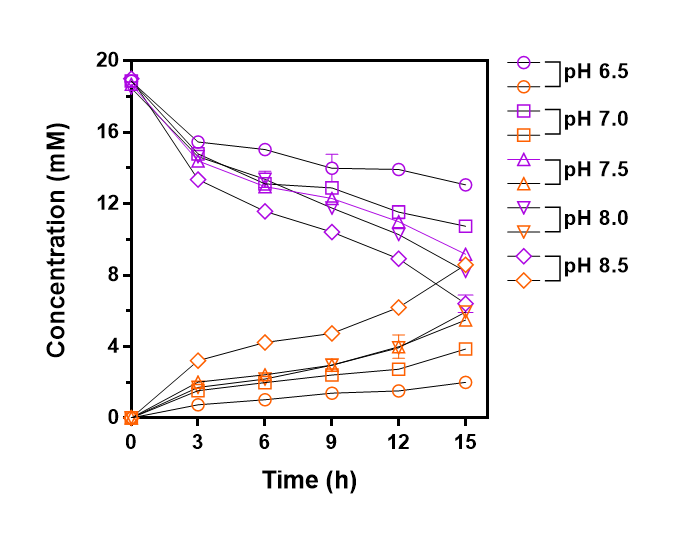


**Figure S2** Evaluation of conversion rate for *t*-CA at various pH conditions in a flask cultivation. Conversion reaction was performed at 30 °C and 18.2 mM of L-Phe was induced. Symbols: open circle, pH 6.5; open square, pH 7.0; open triangle up, pH 7.5; open triangle down, pH 8.0; open diamond, pH 8.5. Purple indicates concentration of L-Phe and orange indicates concentration of *t*-CA. Results are the mean of duplicate experiments and error bars indicates standard deviations.
