# Supplementary material for: Production of trans-cinnamic acid by whole-cell bioconversion from l-phenylalanine in engineered Corynebacterium glutamicum
Source: Microb Cell Fact. 2021 Jul 24;20:145. doi: 10.1186/s12934-021-01631-1 (PMC8310591; doi:10.1186/s12934-021-01631-1)
Supplement: Supplementary file 3 — Additional file 3: Figure S3. Evaluation of bioconversion into t-CA at a bioreactor scale (2 L) with various concentrations of l-Phe as a substrate. [file 12934_2021_1631_MOESM3_ESM.docx]

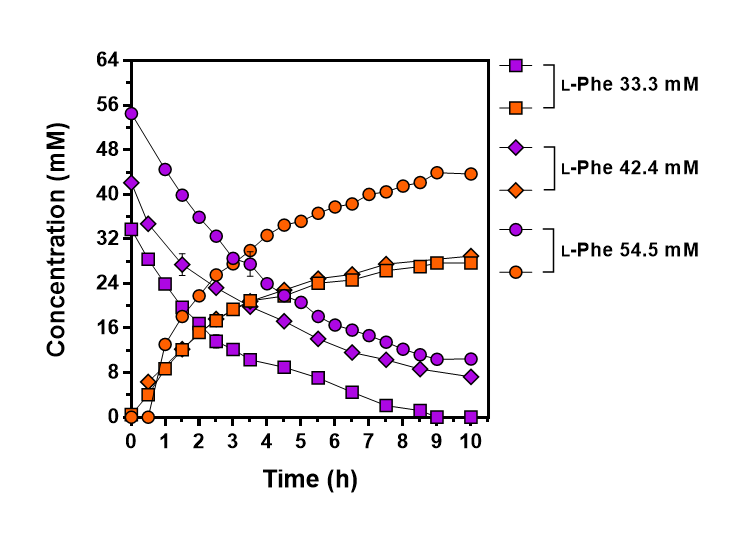


**Figure S3** Evaluation of bioconversion into *t*-CA at a bioreactor scale (2 L) with various concentrations of L-Phe as a substrate. Conversion reaction was performed at pH 8.5 (10 M NaOH) and 50 °C conditions. Symbols: closed square, 33.3 mM of L-Phe; closed diamond, 42.4 mM of L-Phe; closed circle, 54.5 mM of L-Phe. Purple indicates concentration of L-Phe and orange indicates concentration of *t*-CA. Results are the mean of duplicate experiments and error bars indicates standard deviations.
