# Supplementary material for: Production of trans-cinnamic acid by whole-cell bioconversion from l-phenylalanine in engineered Corynebacterium glutamicum
Source: Microb Cell Fact. 2021 Jul 24;20:145. doi: 10.1186/s12934-021-01631-1 (PMC8310591; doi:10.1186/s12934-021-01631-1)
Supplement: Supplementary file 4 — Additional file 4: Table S1. Oligonucleotides used in this study. [file 12934_2021_1631_MOESM4_ESM.docx]

**Table S1 Oligonucleotides used in this study**

| Primer | Sequence (5’ to 3’) |
| --- | --- |
| F-T7R-SmPAL | AATCTAGATAACTTTAAGAAGGAGATATACATATGGGCACCTTCGTTATTGAACT |
| R-SmPAL | CCGCGGCCGCGGCCCCCGAGGCCTCACTTGTCATCGTCATCCTTGT |
